# Clonal hematopoiesis is not prevalent in Hutchinson-Gilford progeria syndrome

**DOI:** 10.1007/s11357-022-00607-2

**Published:** 2022-06-25

**Authors:** Miriam Díez-Díez, Marta Amorós-Pérez, Jorge de la Barrera, Enrique Vázquez, Ana Quintas, Domingo A. Pascual-Figal, Ana Dopazo, Fátima Sánchez-Cabo, Monica E. Kleinman, Leslie B. Gordon, Valentín Fuster, Vicente Andrés, José J. Fuster

**Affiliations:** 1 Centro Nacional de Investigaciones Cardiovasculares (CNIC), Melchor Fernández Almagro, 3., 28029 Madrid, Spain; 2 Centro de Investigacion Biomedica en Red de Enfermedades Cardiovasculares, (CIBERCV) 28029 Madrid, Spain; 3 Hospital Virgen de La Arrixaca, Universidad de Murcia, 30120 Murcia, Spain; 4 Department of Anesthesiology, Critical Care and Pain Medicine, Boston Children’s Hospital and Harvard Medical School, Boston, MA 02115 USA; 5 Department of Pediatrics, Division of Genetics, Hasbro Children’s Hospital and Warren Alpert Medical School of Brown University, Providence, RI 02903 USA; 6 The Progeria Research Foundation, Peabody, MA 01960 USA; 7 Icahn School of Medicine at Mount Sinai, New York, NY 10029 USA

**Keywords:** Somatic mutations, Premature aging syndrome, CHIP, Cardiovascular disease

## Abstract

Clonal hematopoiesis of indeterminate potential (CHIP), defined as the presence of somatic mutations in cancer-related genes in blood cells in the absence of hematological cancer, has recently emerged as an important risk factor for several age-related conditions, especially cardiovascular disease. CHIP is strongly associated with normal aging, but its role in premature aging syndromes is unknown. Hutchinson-Gilford progeria syndrome (HGPS) is an ultra-rare genetic condition driven by the accumulation of a truncated form of the lamin A protein called progerin. HGPS patients exhibit several features of accelerated aging and typically die from cardiovascular complications in their early teens. Previous studies have shown normal hematological parameters in HGPS patients, except for elevated platelets, and low levels of lamin A expression in hematopoietic cells relative to other cell types in solid tissues, but the prevalence of CHIP in HGPS remains unexplored. To investigate the potential role of CHIP in HGPS, we performed high-sensitivity targeted sequencing of CHIP-related genes in blood DNA samples from a cohort of 47 HGPS patients. As a control, the same sequencing strategy was applied to blood DNA samples from middle-aged and elderly individuals, expected to exhibit a biological age and cardiovascular risk profile similar to HGPS patients. We found that CHIP is not prevalent in HGPS patients, in marked contrast to our observations in individuals who age normally. Thus, our study unveils a major difference between HGPS and normal aging and provides conclusive evidence that CHIP is not frequent in HGPS and, therefore, is unlikely to contribute to the pathophysiology of this accelerated aging syndrome.

**Supplementary Information:**

The online version contains supplementary material available at 10.1007/s11357-022-00607-2.

Age-related somatic mutagenesis and genome mosaicism are being actively investigated for its potential causal contribution to human aging and age-related disease [1, 2]. Clonal hematopoiesis of indeterminate potential (CHIP), also known as age-related clonal hematopoiesis (ARCH), is a common condition in which a significant fraction of a cancer-free individual’s blood cells is derived from a single hematopoietic stem cell clone that has a selective advantage due to the acquisition of a somatic mutation in a cancer-related gene. CHIP is strongly associated with chronological age and with indicators of biological age, such as epigenetic clocks [3–5]. Although CHIP typically leads to none or very minor alterations in the absolute counts of circulating blood cells, it has emerged as a potent risk factor for several age-related conditions, most notably cardiovascular disease [3, 6–9]. Sequencing studies in humans and experiments in mice strongly suggest that some CHIP mutations promote the development of atherosclerotic cardiovascular conditions, such as coronary heart disease by exacerbating inflammatory responses [3, 6, 10]. Furthermore, CHIP has also been associated with an adverse clinical progression of heart failure, a leading cause of hospitalization for elderly individuals [7, 9]. Beyond heart disease, some CHIP mutations have been suggested to contribute to the development and progression of a variety of conditions prevalent in the elderly, such as osteoporosis, chronic obstructive pulmonary disease, chronic kidney disease, or insulin resistance [11–14]. However, despite the accumulating evidence supporting the clinical relevance of CHIP in normal aging, its potential contribution to the pathophysiology of accelerated aging syndromes remains unexplored.

Hutchinson-Gilford progeria syndrome (HGPS) is an ultra-rare genetic disease caused by a heterozygous de novo point mutation in the *LMNA* gene (c.1824C > T/p.G608G in most patients) that provokes the production and accumulation of a truncated form of the prelamin A protein called progerin [15]. Progerin expression leads to structural and functional alterations causing genomic instability, increased oxidative stress, and, ultimately, cell senescence [15]. Children with HGPS have normal blood cell profiles, except for elevated platelets [16], and exhibit premature aging and excessive atherosclerosis, with death occurring typically from heart failure or myocardial infarction in their early teens. Human and mouse studies have shown lamin A expression in bone marrow (BM) cells, especially in hematopoietic stem cells [17, 18], although to a lesser extent than in stromal cells. Additionally, lamin A-deficient mice exhibit an aging-like hematopoietic phenotype [17]. Similarly, mice with ubiquitous progerin expression, an experimental model of HGPS, present microenvironmental alterations in BM that lead to hematopoietic stem cell expansion and myeloid-biased differentiation [19]. Furthermore, even though not tested in hematopoietic cells specifically, HGPS is characterized by defective DNA damage repair [20]. Overall, these previous findings suggest that HGPS may facilitate the acquisition of CHIP mutations and/or the expansion of the resulting mutant hematopoietic clones, which in turn could contribute to the age-related features found in these children, including the high cardiovascular risk. Here, we tested this hypothesis by investigating the prevalence of CHIP in HGPS patients.

We performed deep targeted sequencing to assess the presence of CHIP mutations in blood DNA samples from 47 HGPS patients carrying the c.1824C > T/p.G608G mutation, the most frequent HGPS-causing mutation. Age, sex, and causes of death are detailed in Supplementary Table S1. Samples were obtained from The Progeria Research Foundation Cell and Tissue Bank, and DNA was isolated from whole blood. DNA sequencing and CHIP variant calling were performed as in our previous work [9], as detailed in Supplemental Methods. In brief, a custom gene panel was designed to detect the presence of somatic mutations in 12 well-established CHIP-driver genes: *DNMT3A*, *TET2*, *ASXL1*, *JAK2*, *TP53*, *PPM1D*, *IDH2*, *CBL*, *SF3B1*, *SRSF2*, *GNAS*, and *GNB1*, which account for more than 80% of the CHIP driver mutations identified to date [3–6, 9, 21]. Mean base coverage across regions of interest was 3443 before unique molecular identifiers (UMI) family clustering and 3335 after inclusion of UMIs. Common sequencing artifacts and germline mutations were excluded, and candidate CHIP driver mutations were identified based on pre-specified criteria (Supplementary Table S2), previous publications, and in silico pathogenicity predictors, consistent with previous studies [3–6, 9].

As a control, we compared CHIP prevalence in HGPS to that observed in 2 non-HGPS cohorts that can be expected to exhibit biological age and cardiovascular risk profiles comparable to that of the HGPS patients included in this study. These cohorts include 111 healthy middle-aged participants in the Aragon Workers’ Health Study (AWHS) [22] and 62 elderly heart failure (HF) patients [9]. AWHS samples were sequenced de novo for the current study, and HF data were re-analyzed from a previously reported sequencing dataset [9]. Age and gender distribution for the three study cohorts are summarized in Table 1.

**Table 1:**
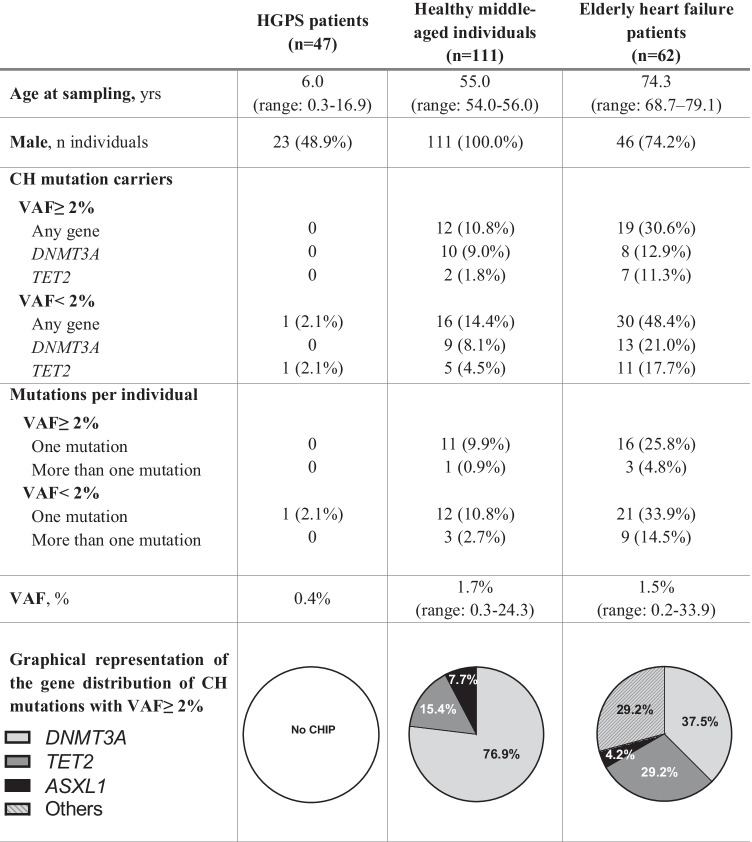
Characteristics and presence of CHIP in HGPS patients, healthy middle-aged individuals, and elderly heart failure patients. Values are median or *n* (%). *CHIP* clonal hematopoiesis of indeterminate potential, *HGPS* Hutchinson-Gilford progeria syndrome. *Any gene* refers to mutations found in *DNMT3A*, *TET2*, *ASXL1*, *JAK2*, *TP53*, *PPM1D*, *IDH2*, *CBL*, *SF3B1*, *SRSF2*, *GNAS*, or *GNB1*

The most frequently used threshold for CHIP definition is a somatic variant allelic fraction (VAF) ≥ 2% (i.e., 4% mutant blood cells, assuming a monoallelic mutation). Using this 2% cutoff, no CHIP-related mutations were identified in HGPS patients (Table 1). Taking advantage of our high-sensitivity sequencing approach, we also assessed the presence of mutations with VAF < 2%. Only a 2-bp deletion affecting the epigenetic modifier *TET2* was found with VAF = 0.4% in one HGPS patient, a 13-year-old female. In marked contrast, for both VAF ≥ 2% and VAF < 2%, CHIP mutations were much more prevalent in healthy middle-aged individuals in the AWHS cohort and in elderly HF patients than in HGPS patients (Table 1, Supplementary Table S3). Most mutations in these normally aging adult populations affected the epigenetic regulators *DNMT3A* and *TET2*, consistent with previous publications [3–5]. A list of all CHIP-driver mutations and their distribution among the three cohorts is included in Supplementary Table S4. Regarding mortality in HGPS patients, 18 out of 47 died at a mean age of 14.6 years, mainly due to cardiovascular-related events (11 out of 18). Blood test in HGPS patients revealed normal leukocyte counts, but increased platelet counts (Supplementary Table S5), consistent with previous human [16] and mouse studies [19].

This study is limited by the modest size of the HGPS cohort. However, HGPS is an ultrarare disease with an estimated prevalence of 1 in 18 million people (https://www.progeriaresearch.org/prf-by-the-numbers/), and the sample size in our study is frequent in HGPS research and typically considered a sufficient representation of the patient population. An important strength of our study is the use of a highly sensitive sequencing strategy, which allows the reliable detection of CHIP mutations even with very low VAFs, in contrast to the whole exome/genome sequencing approaches that are frequently used in CHIP studies [3–6]. This sequencing strategy revealed lack of CHIP in HGPS patients, whereas it did detect a substantial prevalence of CHIP mutations in healthy middle-aged individuals and elderly HF patients, who can be considered adequate biological aging controls for our cohort of HGPS patients. However, as we sequenced canonical CHIP driver genes exclusively, we cannot rule out the possibility that HGPS patients exhibit oligoclonal hematopoiesis driven by unknown drivers, which is emerging as a universal feature of normal aging [23].

Our results demonstrate that CHIP is not prevalent in the setting of premature aging that characterizes HGPS and, therefore, is unlikely to contribute to the accelerated aging phenotype characterized by an increased cardiovascular risk that is observed in patients affected by this progeroid syndrome, in contrast to its emerging role as a potent cardiovascular risk factor in the general population [3, 6]. In a broader context, our findings may reflect that somatic mutagenesis in the hematopoietic system is uncommon at young ages, even in a setting of genomic instability like HGPS, and/or that the progerin-induced senescent phenotype in HGPS patients’ cells [15] may prevent the expansion of hematopoietic clones that acquire CHIP mutations. It is also possible that the development of mutant clones requires a prolonged timeframe, beyond the typical lifespan of HGPS patients, even in conditions of accelerated aging and abnormal hematopoiesis. In agreement with our findings in HGPS children, absence of CHIP with VAF ≥ 2% was also revealed in deep sequencing studies of other young cohorts, including Down syndrome individuals and childhood cancer survivors [24, 25], and only one mutation was identified by whole exome sequencing in a non-cancer cohort of 388 children [26]. These findings reinforce the relevance of time for the expansion of mutant clones that acquire a selective advantage, supporting the association of CHIP with chronological aging and emphasizing the differences between chronological aging (merely reflecting the time passed since birth) and biological aging (the decline over time in tissue and organismal function), which is exaggerated in HGPS patients. Supporting this interpretation, some CHIP mutations have been predicted to be acquired in utero and to require decades to expand to a significant fraction of cells [27]. Overall, our findings provide strong evidence that CHIP is not a shared feature of normal and progeroid aging and highlight the differences between HGPS and chronological aging.

## Supplementary Information

Below is the link to the electronic supplementary material.

Supplementary file1 (DOCX 68 KB)

## Declarations

### Disclaimer

The funders had no role in the design of the study; the collection, analysis, or interpretation of data; the writing of the manuscript; or the decision to publish the results.
