# Supplementary material for: Clonal hematopoiesis is not prevalent in Hutchinson-Gilford progeria syndrome
Source: GeroScience. 2022 Jun 25;45(2):1231–6. doi: 10.1007/s11357-022-00607-2 (PMC9886702; doi:10.1007/s11357-022-00607-2)
Supplement: Supplementary file 1 — Supplementary file1 (DOCX 68 KB) [file 11357_2022_607_MOESM1_ESM.docx]

## Supplemental methods

- **Sample preparation and next-generation sequencing**

DNA was isolated from buffy coat with Maxwell 16 Blood DNA Purification Kit (Promega Corp.). DNA samples were washed with 2x AMPure XP Beads (Beckman Coulter) and 500 ng of each sample were used for library preparation with Kapa HyperPlus Kit (Roche). xGen unique dual-indexes (UDIs) and unique molecular identifiers adapters, also known as UMIs (IDT, Integrated DNA Technologies), were used to improve identification of low-frequency variants and prevent sequencing errors. Libraries were size-selected with AMPure XP Beads to include 450-to-650-bp DNA fragments. A custom gene panel was designed to detect the presence of somatic mutations in 12 well-established CHIP-driver genes: *DNMT3A, TET2, ASXL1, JAK2, TP53, PPM1D, IDH2, CBL, SF3B1, SRSF2, GNAS,* and *GNB1*. A total of 500 ng of each DNA library were used for panel capture. Twelve samples were pooled for each capture and processed as described in the xGen hybridization capture protocol (IDT), using the Kapa HiFi Polymerase (Roche) for PCR amplification. Quality of DNA samples and libraries were confirmed with Nanodrop Spectrophotometer (Thermo Scientific), Qubit Fluorometer (Invitrogen), and Tapestation 2200 (Agilent). Libraries were sequenced on a HiSeq 4000 (Illumina). Read quality was assessed using FastQC.

- **Variant calling and annotation**

Raw sequencing reads were mapped to human genome build GRCh38 using Burrows-Wheeler Alignment–MEM. PCR duplicates were marked based on mapping coordinates and UMIs, and reads from the same fragment were grouped together. Consensus reads for each fragment were obtained and mapped to GRCh38. Bases of consensus reads were masked if base quality was below 10 or differed on opposite strand reads. Putative somatic mutations were identified using Genome Analysis Toolkit (GATK) Mutect2, with gnomAD for the assessment of germline allele fractions, and filtered using GATK. For functional annotation of variants, VCF files of each sample were normalized to split multiallelic loci into biallelic records, and merged into a single VCF file, using bcftools. Variants were then annotated using Variant Effect Predictor.

- **Variant filtering**

Variants were classified as germline if they fulfilled any of the following: (i) were registered with a Minor Allele Frequency ˃1% in 1000Genomes or gnomAD; (ii) had a variant allelic fraction (VAF) between 0.40 and 0.60 or ˃0.80; (iii) were present in more than 10% of samples. Only variants identified as somatic and a functional impact ‘High’ or ‘Moderate’ by Variant Effect Predictor were considered for further analysis. In order to filter out potential artifacts, according to Mutect2 filters, we removed multiallelic somatic calls and variants with deficient mapping or base quality, as well as those flagged by the position filter. In addition, only variants with read depth ≥ 500, alternative depth ≥ 3 and evidence on both forward and reverse strand (F1R2 and F2R1 read pair depth ≥ 1) were considered. Candidate CHIP driver mutations were identified based on pre-specified criteria (**Table S2**), previous publications, presence of ≥3 times in the Catalogue of Somatic Mutations in Cancer (COSMIC, https://cancer.sanger.ac.uk/cosmic) in hematopoietic samples from at least 3 different studies, as well as *in silico* pathogenicity predictors (SIFT and PolyPhen-2). **Supplemental data**

**Table S1.** Characteristics of Hutchinson-Gilford progeria syndrome (HGPS) patients and cause of death.

| Sample | Genetic testing result | Age at donation (years) | Sex | Cause of death |
| --- | --- | --- | --- | --- |
| HGABLDNA095 | Exon 11 c.1824C>T p.G608G | 6,8 | M | CVD-related death |
| HGABLDNA097 | Exon 11 c.1824C>T p.G608G | 2,6 | F | living |
| HGABLDNA110 | Exon 11 c.1824C>T p.G608G | 5,6 | F | stroke |
| HGABLDNA132 | Exon 11 c.1824C>T p.G608G | 1,4 | M | living |
| HGABLDNA145 | Exon 11 c.1824C>T p.G608G | 13 | F | CVD-related death |
| HGABLDNA146 | Exon 11 c.1824C>T p.G608G | 9,2 | F | unknown |
| HGABLDNA150 | Exon 11 c.1824C>T p.G608G | 6 | M | CVD-related death |
| HGABLDNA154 | Exon 11 c.1824C>T p.G608G | 16,9 | M | unknown |
| UHGABLDNA172 | Exon 11 c.1824C>T p.G608G | 7,8 | F | CVD-related death |
| HGABLDNA234-2 | Exon 11 c.1824C>T p.G608G | 10 | F | CVD-related death |
| HGABLDNA236 | Exon 11 c.1824C>T p.G608G | 6,1 | F | CVD-related death |
| HGABLDNA237 | Exon 11 c.1824C>T p.G608G | 14,1 | M | vehicle accident |
| UHGABLDNA240 | Exon 11 c.1824C>T p.G608G | 4,3 | F | living |
| HGABLDNA255 | Exon 11 c.1824C>T p.G608G | 0,8 | F | living |
| HGABLDNA306 | Exon 11 c.1824C>T p.G608G | 2,2 | M | living |
| HGABLDNA314 | Exon 11 c.1824C>T p.G608G | 2,3 | M | living |
| HGABLDNA331 | Exon 11 c.1824C>T p.G608G | 0,8 | M | living |
| HGABLDNA338 | Exon 11 c.1824C>T p.G608G | 3,4 | F | CVD-related death |
| UHGABLDNA351 | Exon 11 c.1824C>T p.G608G | 0,3 | F | living |
| HGABLDNA352 | Exon 11 c.1824C>T p.G608G | 1,5 | F | living |
| HGABLDN377 | Exon 11 c.1824C>T p.G608G | 3 | M | living |
| HGABLDNA378 | Exon 11 c.1824C>T p.G608G | 3,7 | M | CVD-related death |
| UHGABLDNA385 | Exon 11 c.1824C>T p.G608G | 11,4 | M | CVD-related death |
| UHGABLDNA389 | Exon 11 c.1824C>T p.G608G | 8,6 | M | unknown |
| UHGABLDNA395 | Exon 11 c.1824C>T p.G608G | 1,6 | M | living |
| UHGABLDNA408 | Exon 11 c.1824C>T p.G608G | 2,7 | M | living |
| UHGABLDNA419 | Exon 11 c.1824C>T p.G608G | 2 | M | living |
| UHGABLDNA424 | Exon 11 c.1824C>T p.G608G | 15,5 | F | stroke |
| UHGABLDNA439 | Exon 11 c.1824C>T p.G608G | 5,8 | F | living |
| UHGABLDNA451 | Exon 11 c.1824C>T p.G608G | 7,5 | M | living |
| UHGABLDNA452 | Exon 11 c.1824C>T p.G608G | 1,2 | F | living |
| HGABLDNA462 | Exon 11 c.1824C>T p.G608G | 6,2 | M | living |
| HGABLDNA469 | Exon 11 c.1824C>T p.G608G | 9,4 | M | CVD-related death |
| UGHABLDNA471 | Exon 11 c.1824C>T p.G608G | 7,4 | F | CVD-related death |
| UHGABLDNA475 | Exon 11 c.1824C>T p.G608G | 7,8 | F | living |
| HGABLDNA478 | Exon 11 c.1824C>T p.G608G | 3 | M | living |
| UHGABLDNA479 | Exon 11 c.1824C>T p.G608G | 6,6 | F | living |
| UHGABLDNA480 | Exon 11 c.1824C>T p.G608G | 1,4 | M | living |
| UHGABLDNA484 | Exon 11 c.1824C>T p.G608G | 10,9 | M | living |
| UHGABLDNA486 | Exon 11 c.1824C>T p.G608G | 12,3 | F | living |
| UHGABLDNA488 | Exon 11 c.1824C>T p.G608G | 6,1 | F | living |
| UHGABLDAN492 | Exon 11 c.1824C>T p.G608G | 15,9 | M | unknown |
| HGABLDNA503 | Exon 11 c.1824C>T p.G608G | 11 | F | living |
| HGABLDNA505 | Exon 11 c.1824C>T p.G608G | 6,1 | F | living |
| HGABLDNA515 | Exon 11 c.1824C>T p.G608G | 1,7 | M | living |
| HGABLDNA039 | Exon 11 c.1824C>T p.G608G | 3,5 | F | living |
| HGABLDNA092 | Exon 11 c.1824C>T p.G608G | 2,9 | F | living |

**Table S2.** Pre-specified list of criteria used for the identification of candidate clonal hematopoiesis driver variants.

| Gene | RefSeq | Candidate CHIP variants |
| --- | --- | --- |
| *ASXL1* | NM_015338 | Frameshift/nonsense/splice-site in exons 12-13 |
| *CBL* | NM_005188 | Missense in p.349-p.420 (RING finger and linker region) |
| *DNMT3A* | NM_175629 | Frameshift/nonsense/splice-site/ F290I, F290C, missense in p.292 – 350 (PWWP domain), R366P, R366H, R366G, A368T, A368V, R379H, R379C, I407T, I407N, I407S, F414L, F414S, F414C, A462V, K468R, p.482 – 614 (ADD domain), p.634 – 912 (MTase domain) |
| *GNAS* | NM_080425 | R844S, R844C, R844H, R844L, Q870K, Q870R, Q870L, Q870H, R1017C |
| *GNB1* | NM_002074 | K57N, K57M, K57E, K57T, I80T, I80N |
| *IDH2* | NM_002168 | R140W, R140Q, R140L, R140G, R172W, R172G, R172K, R172T, R172M, R172N, R172S |
| *JAK2* | NM_004972 | N533D, N533Y, N533S, H538R, K539E, K539L, I540T, I540V, V617F, R683S, R683G, del/ins537-539L, del/ins538-539L, del/ins540-543MK, del/ins540-544MK, del/ins541-543K, del542-543, del543-544, ins11546-547 |
| *PPM1D* | NM_003620 | Frameshift/nonsense in exons 5-6 |
| *SF3B1* | NM_012433 | G347V, R387W, R387Q, E592K, E622D, Y623C, R625L, R625C, R625G, H662Q, H662D, T663I, K666N, K666T, K666E, K666R, K700E, V701F, A708T, G740R, G740E, K741N, G742D, A744P, D781G, E783K, R831Q, L833F, E862K, R957Q |
| *SRSF2* | NM_003016 | Y44H, P95H, P95L, P95T, P95R, P95A, P107H, P95fs |
| *TET2* | NM_001127208 | Frameshift/nonsense/splice-site, missense mutations in p.1129-1312 (Cys-rich domain), p.1312-1936 (DSBH domain) |
| *TP53* | NM_000546 | Frameshift/nonsense/splice-site, S46F, G105C, G105R, G105D, G108S, G108C, R110L, R110C, T118A, T118R, T118I, S127F, S127Y, L130V, L130F, K132Q, K132E, K132W, K132R, K132M, K132N, F134V. F134L, F134S, C135W, C135S, C135F, C135G, C135Y, Q136K, Q136E, Q136P, Q136R, Q136L, Q136H, A138P, A138V, A138A, A138T, T140I, C141R, C141G, C141A, C141Y, C141S, C141F, C141W, V143M, V143A, V143E, L145Q, W146C, W146L, L145R, V147G, P151T, P151A, P151S, P151H, P151R, P152S, P152R, P152L, T155P, T155A, V157F, R158H, R158L, A159V, A159P, A159S, A159D, A161T, A161D, Y163N, Y163H, Y163D, Y163S, Y163C, K164E, K164M, K164N, K164P, H168Y, H168P, H168R, H168L, H168Q, M169I, M169T, M169V, E171K, E171Q, E171G, E171A, E171V, E171D, V172D, V173M, V173L, V173G, R174W, R175G, R175C, R175H, C176R, C176G, C176Y, C176F, C176S, P177R, P177R, P177L, H178D, H178P, H178Q, H179Y, H179R, H179Q, R181C, R181Y, D186G, G187S, P190L, P190T, H193N, H193P, H193L, H193R, L194F, L194R, I195F, I195N, I195T, R196P, V197L, G199V, Y205N, Y205C, Y205H, D208V, R213Q, R213P, R213L, R213Q, H214D, H214R, S215G, S215I, S215R, V216M, V217G, Y220N, Y220H, Y220S, Y220C, E224D, I232F, I232N, I232T, I232S, Y234N, Y234H, Y234S, Y234C, Y236N, Y236H, Y236C, M237V, M237K, M237I, C238R, C238G, C238Y, C238W, N239T, N239S, S241Y, S241C, S241F, C242G, C242Y, C242S, C242F, G244S, G244C, G244D, G245S, G245R, G245C, G245D, G245A, G245V, G245S, M246V, M246K, M246R, M246I, N247I, R248W, R248G, R248Q, R249G, R249W, R249T, R249M, P250L, I251N, L252P, I254S, I255F, I255N, I255S, L257Q, L257P, E258K, E258Q, D259Y, S261T, G262D, G262V, L265P, G266R, G266E, G266V, R267W, R267Q, R267P, E271K, V272M, V272L, R273S, R273G, R273C, R273H, R273P, R273L, V274F, V274D, V274A, V274G, V274L, C275Y, C275S, C275F, A276P, C277F, C277Y, P278T, P278A, P278S, P278H, P278R, P278L, G279E, R280G, R280K, R280T, R280I, R280S, D281N, D281H, D281Y, D281G, D281E, R282G, R282W, R282Q, R282P, E285K, E285V, E286G, E286V, E286K, K320N, L330R, G334V, R337C, R337L, A347T, L348F, T377P |

**Table S3.** One-sided p-value of the proportions test using the prop.test function from the stats R package is reported, being the alternative to the null hypothesis that the prevalence of CHIP in the middle-aged healthy group or in the elderly group is larger than in the HGPS group. CHIP, clonal hematopoiesis of indeterminate potential; HGPS, Hutchinson-Gilford progeria syndrome; VAF, variant allelic fraction.

|  | HGPS vs  Healthy middle-aged | HGPS vs  Elderly heart failure |
| --- | --- | --- |
| Prevalence of CHIP with VAF≥ 2% (Any gene) | 0.022 | 4.396x10^-5^ |
| Prevalence of CHIP with VAF< 2% (Any gene) | 0.023 | 1.814x10^-7^ |

**Table S4.** List of variants identified as drivers of clonal hematopoiesis in Hutchinson-Gilford progeria syndrome (HGPS) patients, healthy middle-aged individuals (Aragon Workers Health Study, AWHS) and elderly heart failure patients (HF).

| Cohort | Sample | Gene | Chr | Position | Ref. allele | Alt. Allele | Ref. reads | Alt. Reads | VAF  (%) | Variant class | HGVSc | HGVSp | Consequence |
| --- | --- | --- | --- | --- | --- | --- | --- | --- | --- | --- | --- | --- | --- |
| HGPS | HGABLDNA145 | ***TET2*** | 4 | 105234566 | TAA | T | 5112 | 22 | 0,004 | deletion | NM_001127208.2: c.625_626del | NP_001120680.1: p.Asn209TrpfsTer15 | frameshift |
| AWHS | B_01 | ***ASXL1*** | 20 | 32434867 | G | GAAGGC | 2877 | 9 | 0,003 | insertion | NM_015338.6: c.2156_2157insAGGCA | NP_056153.2: p.Asp720GlyfsTer7 | frameshift |
| AWHS | B_02 | ***ASXL1*** | 20 | 32437256 | TGTGCCAA | T | 2997 | 7 | 0,003 | deletion | NM_015338.6: c.4546_4552del | NP_056153.2: p.Cys1516AlafsTer23 | frameshift |
| AWHS | B_03 | ***ASXL1*** | 20 | 32435587 | CT | C | 4058 | 43 | 0,011 | deletion | NM_015338.6: c.2876del | NP_056153.2: p.Leu959ProfsTer25 | frameshift |
| AWHS | B_04 | ***ASXL1*** | 20 | 32433839 | C | CT | 3026 | 229 | 0,069 | insertion | NM_015338.6: c.1644dup | NP_056153.2: p.Arg549SerfsTer2 | frameshift |
| AWHS | B_01 | ***DNMT3A*** | 2 | 25235726 | A | G | 626 | 11 | 0,016 | SNV | NM_175629.2: c.2578T>C | NP_783328.1: p.Trp860Arg | missense |
| AWHS | B_01 | ***DNMT3A*** | 2 | 25247722 | G | C | 1683 | 21 | 0,013 | SNV | NM_175629.2: c.883C>G | NP_783328.1: p.Leu295Val | missense |
| AWHS | B_07 | ***DNMT3A*** | 2 | 25248113 | GT | G | 1896 | 8 | 0,005 | deletion | NM_175629.2: c.778del | NP_783328.1: p.Thr260ProfsTer56 | frameshift |
| AWHS | B_07 | ***DNMT3A*** | 2 | 25246241 | G | GAACC | 2023 | 6 | 0,003 | insertion | NM_175629.2: c.1347_1348insGGTT | NP_783328.1: p.Pro450GlyfsTer24 | frameshift |
| AWHS | B_09 | ***DNMT3A*** | 2 | 25236958 | A | T | 3163 | 29 | 0,009 | SNV | NM_175629.2: c.2456T>A | NP_783328.1: p.Leu819Gln | missense |
| AWHS | B_10 | ***DNMT3A*** | 2 | 25234389 | CGTCAGTATAGT | C | 3594 | 12 | 0,004 | deletion | NM_175629.2: c.2618_2628del | NP_783328.1: p.His873ArgfsTer44 | frameshift |
| AWHS | B_11 | ***DNMT3A*** | 2 | 25241704 | A | T | 3129 | 59 | 0,017 | SNV | NM_175629.2: c.1940T>A | NP_783328.1: p.Leu647His | missense |
| AWHS | B_12 | ***DNMT3A*** | 2 | 25240326 | CT | C | 3946 | 42 | 0,010 | deletion | NM_175629.2: c.2297del | NP_783328.1: p.Lys766ArgfsTer13 | frameshift |
| AWHS | B_13 | ***DNMT3A*** | 2 | 25234374 | G | A | 4016 | 30 | 0,007 | SNV | NM_175629.2: c.2644C>T | NP_783328.1: p.Arg882Cys | missense |
| AWHS | B_09 | ***DNMT3A*** | 2 | 25241709 | T | C | 3353 | 48 | 0,014 | SNV | NM_175629.2: c.1937-2A>G |  | splice_acceptor |
| AWHS | B_15 | ***DNMT3A*** | 2 | 25244214 | G | A | 4593 | 46 | 0,010 | SNV | NM_175629.2: c.1792C>T | NP_783328.1: p.Arg598Ter | stop_gained |
| AWHS | B_16 | ***DNMT3A*** | 2 | 25240326 | CTT | C | 5043 | 33 | 0,007 | deletion | NM_175629.2: c.2296_2297del | NP_783328.1: p.Lys766GlufsTer15 | frameshift |
| AWHS | B_17 | ***DNMT3A*** | 2 | 25247160 | T | C | 1212 | 355 | 0,226 | SNV | NM_175629.2: c.1015-2A>G |  | splice_acceptor |
| AWHS | B_18 | ***DNMT3A*** | 2 | 25241668 | C | T | 1378 | 177 | 0,111 | SNV | NM_175629.2: c.1976G>A | NP_783328.1: p.Arg659His | missense |
| AWHS | B_19 | ***DNMT3A*** | 2 | 25247157 | A | AC | 2051 | 41 | 0,020 | insertion | NM_175629.2: c.1015dup | NP_783328.1: p.Val339GlyfsTer4 | frameshift& splice_region |
| AWHS | B_20 | ***DNMT3A*** | 2 | 25234374 | G | A | 3066 | 991 | 0,243 | SNV | NM_175629.2: c.2644C>T | NP_783328.1: p.Arg882Cys | missense |
| AWHS | B_21 | ***DNMT3A*** | 2 | 25240708 | T | C | 3066 | 70 | 0,021 | SNV | NM_175629.2: c.2105A>G | NP_783328.1: p.Asp702Gly | missense |
| AWHS | B_22 | ***DNMT3A*** | 2 | 25234373 | C | G | 3284 | 244 | 0,068 | SNV | NM_175629.2: c.2645G>C | NP_783328.1: p.Arg882Pro | missense |
| AWHS | B_23 | ***DNMT3A*** | 2 | 25246733 | TC | T | 3382 | 84 | 0,024 | deletion | NM_175629.2: c.1165del | NP_783328.1: p.Asp389ThrfsTer18 | frameshift |
| AWHS | B_24 | ***DNMT3A*** | 2 | 25240373 | A | C | 3509 | 202 | 0,054 | SNV | NM_175629.2: c.2251T>G | NP_783328.1: p.Phe751Val | missense |
| AWHS | B_25 | ***DNMT3A*** | 2 | 25246682 | AT | A | 4164 | 156 | 0,036 | deletion | NM_175629.2: c.1216del | NP_783328.1: p.Met406Ter | frameshift |
| AWHS | B_26 | ***DNMT3A*** | 2 | 25234374 | G | A | 4295 | 101 | 0,022 | SNV | NM_175629.2: c.2644C>T | NP_783328.1: p.Arg882Cys | missense |
| AWHS | B_27 | ***TET2*** | 4 | 105235968 | CTGTG | C | 3458 | 58 | 0,017 | deletion | NM_001127208.2: c.2029_2032del | NP_001120680.1: p.Cys677AlafsTer22 | frameshift |
| AWHS | B_28 | ***TET2*** | 4 | 105237352 | G | GT | 4092 | 71 | 0,017 | insertion | NM_001127208.2: c.3409+2dup |  | splice_donor |
| AWHS | B_29 | ***TET2*** | 4 | 105235275 | CT | C | 4621 | 86 | 0,018 | deletion | NM_001127208.2: c.1337del | NP_001120680.1: p.Leu446Ter | frameshift |
| AWHS | B_30 | ***TET2*** | 4 | 105272882 | CAA | C | 4299 | 23 | 0,005 | deletion | NM_001127208.2: c.4503_4504del | NP_001120680.1: p.Gln1501HisfsTer2 | frameshift |
| AWHS | B_31 | ***TET2*** | 4 | 105234591 | TC | T | 6344 | 40 | 0,006 | deletion | NM_001127208.2: c.651del | NP_001120680.1: p.Val218TrpfsTer32 | frameshift |
| AWHS | B_18 | ***TET2*** | 4 | 105259666 | C | T | 2040 | 79 | 0,035 | SNV | NM_001127208.2: c.3851C>T | NP_001120680.1: p.Ser1284Phe | missense |
| AWHS | B_33 | ***TET2*** | 4 | 105269662 | G | A | 2856 | 210 | 0,068 | SNV | NM_001127208.2: c.4097G>A | NP_001120680.1: p.Arg1366His | missense |
| HF | C_01 | ***ASXL1*** | 20 | 32434474 | C | T | 4761 | 59 | 0,011 | SNV | NM_015338.6: c.1762C>T | NP_056153.2: p.Gln588Ter | stop_gained |
| HF | C_02 | ***ASXL1*** | 20 | 32434871 | AC | A | 5371 | 57 | 0,010 | deletion | NM_015338.6: c.2161del | NP_056153.2: p.Leu721CysfsTer4 | frameshift |
| HF | C_03 | ***ASXL1*** | 20 | 32435141 | A | AC | 5716 | 87 | 0,014 | insertion | NM_015338.6: c.2430dup | NP_056153.2: p.Asn811GlnfsTer11 | frameshift |
| HF | C_04 | ***ASXL1*** | 20 | 32436269 | CT | C | 5528 | 93 | 0,017 | deletion | NM_015338.6: c.3558del | NP_056153.2: p.Gly1187ValfsTer30 | frameshift |
| HF | C_04 | ***ASXL1*** | 20 | 32435174 | ATACAT | A | 5543 | 213 | 0,038 | deletion | NM_015338.6: c.2465_2469del | NP_056153.2: p.Thr822ArgfsTer9 | frameshift |
| HF | C_06 | ***CBL*** | 11 | 119278236 | A | C | 2570 | 111 | 0,042 | SNV | NM_005188.4: c.1166A>C | NP_005179.2: p.Lys389Thr | missense |
| HF | C_07 | ***DNMT3A*** | 2 | 25234416 | ATACCCTGG | A | 4458 | 6 | 0,002 | deletion | NM_175629.2: c.2598-4_2601del |  | splice_acceptor&coding_sequence&intron |
| HF | C_08 | ***DNMT3A*** | 2 | 25240349 | CCA | C | 4273 | 17 | 0,004 | deletion | NM_175629.2: c.2273_2274del | NP_783328.1: p.Val758GlyfsTer6 | frameshift |
| HF | C_09 | ***DNMT3A*** | 2 | 25245269 | ATTCCTCCAAC | A | 2437 | 7 | 0,004 | deletion | NM_175629.2: c.1528_1537del | NP_783328.1: p.Val510CysfsTer138 | frameshift |
| HF | C_10 | ***DNMT3A*** | 2 | 25246200 | C | CT | 4685 | 64 | 0,013 | insertion | NM_175629.2: c.1388dup | NP_783328.1: p.Lys464GlufsTer9 | frameshift |
| HF | C_11 | ***DNMT3A*** | 2 | 25246660 | GC | G | 5326 | 54 | 0,009 | deletion | NM_175629.2: c.1238del | NP_783328.1: p.Gly413AlafsTer238 | frameshift |
| HF | C_12 | ***DNMT3A*** | 2 | 25246773 | CCACCTGGAGGGTGACA | C | 5239 | 9 | 0,002 | deletion | NM_175629.2: c.1123-13_1125del |  | splice_acceptor&coding_sequence&intron |
| HF | C_13 | ***DNMT3A*** | 2 | 25248132 | C | CT | 4035 | 35 | 0,008 | insertion | NM_175629.2: c.759_760insA | NP_783328.1: p.Ala254SerfsTer10 | frameshift |
| HF | C_14 | ***DNMT3A*** | 2 | 25240400 | G | C | 5652 | 39 | 0,007 | SNV | NM_175629.2: c.2224C>G | NP_783328.1: p.Arg742Gly | missense |
| HF | C_15 | ***DNMT3A*** | 2 | 25240639 | C | T | 4389 | 51 | 0,010 | SNV | NM_175629.2: c.2173+1G>A |  | splice_donor |
| HF | C_16 | ***DNMT3A*** | 2 | 25234308 | G | A | 4786 | 68 | 0,013 | SNV | NM_175629.2: c.2710C>T | NP_783328.1: p.Pro904Ser | missense |
| HF | C_14 | ***DNMT3A*** | 2 | 25247628 | C | T | 5389 | 88 | 0,015 | SNV | NM_175629.2: c.977G>A | NP_783328.1: p.Arg326His | missense |
| HF | C_18 | ***DNMT3A*** | 2 | 25241587 | T | C | 4257 | 78 | 0,017 | SNV | NM_175629.2: c.2057A>G | NP_783328.1: p.Asp686Gly | missense |
| HF | C_03 | ***DNMT3A*** | 2 | 25241669 | G | A | 4363 | 78 | 0,017 | SNV | NM_175629.2: c.1975C>T | NP_783328.1: p.Arg659Cys | missense |
| HF | C_20 | ***DNMT3A*** | 2 | 25244540 | C | G | 2857 | 57 | 0,019 | SNV | NM_175629.2: c.1667G>C | NP_783328.1: p.Arg556Thr | missense& splice_region |
| HF | C_20 | ***DNMT3A*** | 2 | 25246051 | G | C | 4532 | 83 | 0,019 | SNV | NM_175629.2: c.1443C>G | NP_783328.1: p.Tyr481Ter | stop_gained |
| HF | C_22 | ***DNMT3A*** | 2 | 25235726 | A | G | 2392 | 109 | 0,044 | SNV | NM_175629.2: c.2578T>C | NP_783328.1: p.Trp860Arg | missense |
| HF | C_23 | ***DNMT3A*** | 2 | 25240315 | G | C | 4911 | 108 | 0,021 | SNV | NM_175629.2: c.2309C>G | NP_783328.1: p.Ser770Trp | missense |
| HF | C_24 | ***DNMT3A*** | 2 | 25240353 | A | T | 4941 | 313 | 0,059 | SNV | NM_175629.2: c.2271T>A | NP_783328.1: p.Asn757Lys | missense |
| HF | C_25 | ***DNMT3A*** | 2 | 25240439 | G | A | 3625 | 1060 | 0,227 | SNV | NM_175629.2: c.2185C>T | NP_783328.1: p.Arg729Trp | missense |
| HF | C_26 | ***DNMT3A*** | 2 | 25241700 | CA | AG | 1112 | 27 | 0,025 | substitution | NM_175629.2: c.1943_1944delinsCT | NP_783328.1: p.Leu648Pro | missense |
| HF | C_16 | ***DNMT3A*** | 2 | 25246165 | GT | G | 4786 | 455 | 0,087 | deletion | NM_175629.2: c.1423del | NP_783328.1: p.Thr475GlnfsTer176 | frameshift |
| HF | C_28 | ***DNMT3A*** | 2 | 25246245 | G | C | 4374 | 197 | 0,042 | SNV | NM_175629.2: c.1344C>G | NP_783328.1: p.Tyr448Ter | stop_gained |
| HF | C_03 | ***DNMT3A*** | 2 | 25247722 | G | C | 2814 | 1451 | 0,339 | SNV | NM_175629.2: c.883C>G | NP_783328.1: p.Leu295Val | missense |
| HF | C_23 | ***DNMT3A*** | 2 | 25248183 | G | A | 4341 | 290 | 0,063 | SNV | NM_175629.2: c.709C>T | NP_783328.1: p.Gln237Ter | stop_gained |
| HF | C_14 | ***GNB1*** | 1 | 1815790 | T | C | 4340 | 51 | 0,011 | SNV | NM_002074.5: c.169A>G | NP_002065.1: p.Lys57Glu | missense |
| HF | C_32 | ***GNB1*** | 1 | 1815790 | T | C | 2043 | 580 | 0,222 | SNV | NM_002074.5: c.169A>G | NP_002065.1: p.Lys57Glu | missense |
| HF | C_03 | ***JAK2*** | 9 | 5073770 | G | T | 3263 | 294 | 0,081 | SNV | NM_004972.3: c.1849G>T | NP_004963.1: p.Val617Phe | missense |
| HF | C_34 | ***PPM1D*** | 17 | 60663169 | GCTAAAGCC | G | 4504 | 22 | 0,005 | deletion | NM_003620.4: c.1438_1445del | NP_003611.1: p.Lys480AspfsTer6 | frameshift |
| HF | C_35 | ***PPM1D*** | 17 | 60663169 | GCTAAAGCC | G | 4137 | 22 | 0,005 | deletion | NM_003620.4: c.1438_1445del | NP_003611.1: p.Lys480AspfsTer6 | frameshift |
| HF | C_22 | ***PPM1D*** | 17 | 60663251 | CT | C | 4500 | 13 | 0,003 | deletion | NM_003620.4: c.1518del | NP_003611.1: p.Val507SerfsTer7 | frameshift |
| HF | C_37 | ***PPM1D*** | 17 | 60663168 | C | A | 4485 | 88 | 0,018 | SNV | NM_003620.4: c.1434C>A | NP_003611.1: p.Cys478Ter | stop_gained |
| HF | C_03 | ***SF3B1*** | 2 | 197402110 | T | C | 3333 | 18 | 0,006 | SNV | NM_012433.3: c.2098A>G | NP_036565.2: p.Lys700Glu | missense |
| HF | C_39 | ***SF3B1*** | 2 | 197401985 | C | G | 3466 | 28 | 0,008 | SNV | NM_012433.3: c.2223G>C | NP_036565.2: p.Lys741Asn | missense& splice_region |
| HF | C_25 | ***SF3B1*** | 2 | 197402110 | T | C | 2275 | 608 | 0,211 | SNV | NM_012433.3: c.2098A>G | NP_036565.2: p.Lys700Glu | missense |
| HF | C_41 | ***SRSF2*** | 17 | 76736877 | G | T | 4678 | 15 | 0,002 | SNV | NM_003016.4: c.284C>A | NP_003007.2: p.Pro95His | missense |
| HF | C_01 | ***SRSF2*** | 17 | 76736877 | G | C | 2406 | 84 | 0,034 | SNV | NM_003016.4: c.284C>G | NP_003007.2: p.Pro95Arg | missense |
| HF | C_15 | ***TET2*** | 4 | 105234566 | TAA | T | 6099 | 17 | 0,003 | deletion | NM_001127208.2: c.625_626del | NP_001120680.1: p.Asn209TrpfsTer15 | frameshift |
| HF | C_44 | ***TET2*** | 4 | 105234882 | TG | T | 5779 | 82 | 0,014 | deletion | NM_001127208.2: c.941del | NP_001120680.1: p.Cys314PhefsTer33 | frameshift |
| HF | C_45 | ***TET2*** | 4 | 105235397 | GA | G | 5858 | 64 | 0,010 | deletion | NM_001127208.2: c.1457del | NP_001120680.1: p.Asn486ThrfsTer11 | frameshift |
| HF | C_08 | ***TET2*** | 4 | 105236094 | CT | C | 5422 | 24 | 0,004 | deletion | NM_001127208.2: c.2156del | NP_001120680.1: p.Leu719CysfsTer32 | frameshift |
| HF | C_47 | ***TET2*** | 4 | 105275470 | C | T | 5636 | 38 | 0,006 | SNV | NM_001127208.2: c.4960C>T | NP_001120680.1: p.Gln1654Ter | stop_gained |
| HF | C_48 | ***TET2*** | 4 | 105236527 | T | TG | 5164 | 23 | 0,005 | insertion | NM_001127208.2: c.2586dup | NP_001120680.1: p.His863AlafsTer9 | frameshift |
| HF | C_25 | ***TET2*** | 4 | 105237196 | C | CA | 5234 | 35 | 0,006 | insertion | NM_001127208.2: c.3256dup | NP_001120680.1: p.Thr1086AsnfsTer18 | frameshift |
| HF | C_18 | ***TET2*** | 4 | 105237262 | C | CA | 5206 | 55 | 0,011 | insertion | NM_001127208.2: c.3321dup | NP_001120680.1: p.Pro1108ThrfsTer22 | frameshift |
| HF | C_22 | ***TET2*** | 4 | 105276160 | A | G | 5254 | 58 | 0,010 | SNV | NM_001127208.2: c.5650A>G | NP_001120680.1: p.Thr1884Ala | missense |
| HF | C_52 | ***TET2*** | 4 | 105243649 | TG | T | 4398 | 41 | 0,009 | deletion | NM_001127208.2: c.3675del | NP_001120680.1: p.Ile1226LeufsTer2 | frameshift |
| HF | C_25 | ***TET2*** | 4 | 105269626 | GA | G | 4460 | 46 | 0,010 | deletion | NM_001127208.2: c.4062del | NP_001120680.1: p.Ala1355HisfsTer8 | frameshift |
| HF | C_24 | ***TET2*** | 4 | 105235590 | C | T | 5277 | 89 | 0,016 | SNV | NM_001127208.2: c.1648C>T | NP_001120680.1: p.Arg550Ter | stop_gained |
| HF | C_55 | ***TET2*** | 4 | 105236688 | C | T | 6120 | 147 | 0,023 | SNV | NM_001127208.2: c.2746C>T | NP_001120680.1: p.Gln916Ter | stop_gained |
| HF | C_56 | ***TET2*** | 4 | 105237193 | A | C | 5775 | 212 | 0,035 | SNV | NM_001127208.2: c.3251A>C | NP_001120680.1: p.Gln1084Pro | missense |
| HF | C_41 | ***TET2*** | 4 | 105242922 | A | G | 3973 | 139 | 0,034 | SNV | NM_001127208.2: c.3589A>G | NP_001120680.1: p.Lys1197Glu | missense |
| HF | C_58 | ***TET2*** | 4 | 105243759 | C | T | 4244 | 86 | 0,021 | SNV | NM_001127208.2: c.3784C>T | NP_001120680.1: p.Arg1262Trp | missense |
| HF | C_03 | ***TET2*** | 4 | 105269703 | C | T | 3396 | 1675 | 0,328 | SNV | NM_001127208.2: c.4138C>T | NP_001120680.1: p.His1380Tyr | missense |
| HF | C_60 | ***TET2*** | 4 | 105275075 | TG | T | 4368 | 171 | 0,038 | deletion | NM_001127208.2: c.4566del | NP_001120680.1: p.Met1522IlefsTer49 | frameshift |
| HF | C_61 | ***TET2*** | 4 | 105275662 | G | T | 5412 | 234 | 0,041 | SNV | NM_001127208.2: c.5152G>T | NP_001120680.1: p.Val1718Leu | missense |
| HF | C_62 | ***TP53*** | 17 | 7674238 | C | T | 1878 | 20 | 0,010 | SNV | NM_000546.5: c.725G>A | NP_000537.3: p.Cys242Tyr | missense |
| HF | C_02 | ***TP53*** | 17 | 7674221 | G | A | 1937 | 37 | 0,017 | SNV | NM_000546.5: c.742C>T | NP_000537.3: p.Arg248Trp | missense |
| HF | C_03 | ***TP53*** | 17 | 7675086 | A | T | 4630 | 114 | 0,023 | SNV | NM_000546.5: c.526T>A | NP_000537.3: p.Cys176Ser | missense |
| HF | C_65 | ***TP53*** | 17 | 7675088 | C | T | 3449 | 779 | 0,184 | SNV | NM_000546.5: c.524G>A | NP_000537.3: p.Arg175His | missense |

Note: Chr., Chromosome; Ref., reference; Alt., Alternate; SNV, single nucleotide variant; VAF, variant allele fraction.

**Table S5: Cell blood counts in children with HGPS.** Values are median (range), and reference ranges in non-HGPS children are shown (normal range). Cell blood counts were available for 35 individuals.

| Variable | Median (range) in HGPS patients | Reference range in healthy children |
| --- | --- | --- |
| WBC (K cells/uL) | 9.3 (5.9- 20.5) | 6.0- 17.5 |
| Hemoglobin (g/dL) | 12.9 (10.0- 14.9) | 10.0- 15.4 |
| Hematocrit (%) | 37.7 (31.9- 44.6) | 30.0- 45.0 |
| Platelets (K cells/uL) | 422 (242- 832) | 150- 350 |
| Prothrombin times (sec x 10^-3^) | 11.5 (10.0- 17.4) | 10.6- 14.7 |
| RBC (M cells/uL) | 4.8 (4.1- 6.4) | 3.8- 5.2 |
| Absolute neutrophil count (K cells/uL) | 3.9 (0.7- 10.0) | 0.5- 7.5 |
| Absolute lymphocyte count (K cells/uL) | 4.2 (2.5- 7.9) | 1.5- 8.5 |
| Absolute monocyte count (K cells/uL) | 0.6 (0.1- 1.4) | 0.2- 1.0 |
| Neutrophils (%) | 41.9 (11.6- 70.0) | 15.0- 72.0 |
| Lymphocyte (%) | 44.3 (23.0- 66.0) | 25.0- 77.0 |
| Monocyte (%) | 5.4 (1.0- 10.1) | 4.0- 10.0 |
| Eosinophil (%) | 3.0 (0- 13.0) | 0- 3.0 |
| Basophil (%) | 1.0 (0- 2.0) | 0- 1.0 |
| Atypical lymphocyte (%) | 3.0 (1.0- 15.3) | 0 |

Note: HGPS, Hutchinson-Gilford progeria syndrome; WBC, white blood cells; RBC, red blood cells.
